# Arimoclomol in infants with Niemann-Pick disease type C: Results from the phase 2/3 open-label pediatric substudy

**DOI:** 10.1016/j.ymgmr.2026.101332

**Published:** 2026-06-19

**Authors:** Eugen Mengel, Laila Arash-Kaps, Stephanie Grunewald, Sabine Weller Grønborg, Natalie Berger, Hadeel Shammas, Christine í Dali

**Affiliations:** a SphinCS, Institute of Clinical Science for LSD, Hochheim, Germany; b Department of Metabolic Medicine, Great Ormond Street Hospital for Children NHS Foundation Trust, NIHR Biomedical Research Centre, London, UK; c Center for Inherited Metabolic Diseases, Department of Pediatrics and Adolescent Medicine and Department of Clinical Genetics, Copenhagen University Hospital Rigshospitalet, Copenhagen, Denmark; d Psychiatry and Psychology Services, Rush University Medical Center, Chicago, IL, USA; e Zevra Therapeutics, Copenhagen, Denmark

**Keywords:** Arimoclomol, Development, Infants, Niemann-Pick disease type C, Open-label, Pharmacokinetics, Safety

## Abstract

**Background:**

Arimoclomol has been approved in the US for the treatment of Niemann-Pick disease type C (NPC) in patients aged ≥2 years, in combination with miglustat. This multicenter, open-label substudy of the phase 2/3 NPC-002 trial (NCT02612129) evaluated the safety, pharmacokinetics (PK) and impact on clinical status outcomes of arimoclomol in infants with NPC 6–<24 months of age.

**Methods:**

Infants with NPC aged 6–<24 months received arimoclomol in addition to their standard of care management for up to 36 months. The dosing regimen used for patients <24 months differed from the regimen recommended in the FDA label. The primary endpoint was safety and tolerability of arimoclomol; secondary endpoints were changes in clinical status (physical examination and Bayley III developmental scores), biomarkers, and PK.

**Results:**

Five patients (three females, two males; aged 14–23 months at screening) were enrolled; four remained in the study >12 months; arimoclomol exposure ranged from 72 to 1109 days. All patients received concomitant miglustat. Across 108 reported adverse events (AEs), most were considered mild or moderate in severity and non-serious. A total of 15 serious AEs were reported for two patients. Two AEs in one patient (elevated alanine/aspartate aminotransferases) were considered probably related to arimoclomol and resolved within 51 days; the patient was withdrawn from the substudy. No clinically significant changes were observed in hematology, kidney ultrasound imaging, or vital signs. Mean arimoclomol exposure over the first 8 h post-dose (1378.3–2988 h∙μg/L) was comparable to levels in NPC patients aged 2–19 years. Changes in Bayley III scores and biomarkers varied between individuals.

**Conclusion:**

Arimoclomol was well tolerated in infants initiating treatment before 2 years of age, with no new safety signals. PK profiles support the dosing regimen used. These findings suggest that early initiation of arimoclomol could be considered for the 6–24-month population. Further investigation in larger cohorts is warranted to elucidate the impact of arimoclomol in NPC patients under 2 years of age.

## 1 Introduction

Niemann-Pick disease type C (NPC) is an ultra-rare disorder with autosomal recessive inheritance, primarily affecting the nervous system and internal organs. The disease results from pathogenic variants in both alleles of either the *NPC1* (in about 95% of cases) or the *NPC2* gene (approximately 5%) which encode for proteins involved in the transport and processing of lipids, such as cholesterol, within the *endo*-lysosomal system. Disrupted function of NPC1 or NPC2 results in cholesterol mistrafficking and accumulation of multiple lipid species such as unesterified cholesterol and a broad range of sphingolipids, causing the neurodegeneration and peripheral organ impairment associated with NPC [1], [2], [3], [4].

Clinically, NPC presents with a broad spectrum of symptoms that worsen over time, including coordination problems (cerebellar ataxia), dysarthria and swallowing issues, cognitive decline, and vertical supranuclear gaze palsy. The onset and rate of progression of NPC vary widely, ranging from severe forms with onset in infancy to more gradual neurodegeneration in adulthood. The disease course is highly individual and often non-linear [2], [5].

Arimoclomol (MIPLYFFA®, Zevra Therapeutics) is an orally available small molecule capable of crossing the blood-brain barrier [6], [7]. It functions by stimulating the transcription factors TFEB and TFE3, which in turn trigger the expression of genes associated with the Coordinated Lysosomal Expression and Regulation (CLEAR) network, including the *NPC1* gene. Activation of the CLEAR network is thought to enhance lysosomal performance and decrease the accumulation of cholesterol within lysosomes [6].

The efficacy and safety of arimoclomol in patients with NPC have been established in the phase 2/3 CT-ORZY-NPC-002 trial (further referred to as the NPC-002 trial; ClinicalTrials.gov identifier: NCT02612129). In the 12-month double-blind phase, arimoclomol in combination with miglustat slowed disease progression through 12 months of treatment [8]. Safety results showed good tolerability. The subsequent open-label extension phase of the study showed a sustained reduction in disease progression for at least 5 years with no new safety concerns for patients receiving arimoclomol in combination with miglustat [9]. Based on the results of the NPC-002 study, arimoclomol recently received US Food and Drug Administration (FDA) approval for use in combination with miglustat (a glucosylceramide synthase inhibitor) for the treatment of neurological manifestations of NPC in adult and pediatric patients 2 years or age and older.

Treatment options for children with NPC under 2 years of age remain limited. Aside from miglustat, which is approved in Europe and several non-US countries for this age group, no disease-modifying therapies are currently available. While miglustat has shown modest long-term benefit in slowing disease progression in clinical trials [10], [11], [12], studies have shown little benefit in young children with the early-infantile form of NPC, underlining the need for additional therapeutic options [13], [14].

This paper presents safety, pharmacokinetic (PK), and therapeutic response data from a multicenter, open-label substudy of arimoclomol in infants aged 6 to <24 months at the time of enrolment.

## 2 Methods

### 2.1 Study objectives

The NPC-002 clinical trial was expanded to incorporate a pediatric substudy after review of juvenile toxicology data and PK profiles from all enrolled patients in the trial aged 2 to <12 years. This expansion was part of the Pediatric Investigation Plan agreement between Zevra and the European Medicines Agency (EMA).

The primary objective of the pediatric substudy was to evaluate the safety and tolerability of arimoclomol in patients aged 6 to <24 months at study enrolment. Additional objectives were to evaluate the therapeutic response and the PK profile of arimoclomol in this patient population.

### 2.2 Study design

The pediatric substudy was a phase 2/3 multicenter, open-label study including patients with a confirmed diagnosis of NPC1 or NPC2 aged 6 to <24 months at study enrolment. The study was performed at sites who participated in the NPC-002 study.

**Fig. 1:**
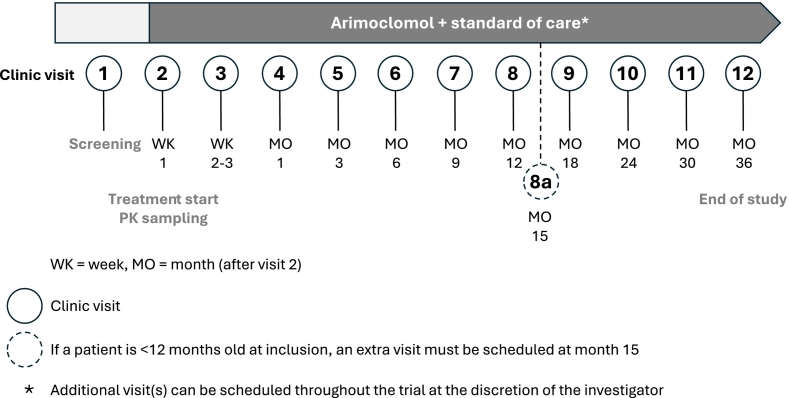
Study design.

After a one-week screening period, all patients received arimoclomol in addition to their standard of care management, which included miglustat. Patients were followed up at regular timepoints over the 36-month trial period (Fig. 1). Additionally, telephone contact between the investigator and the patients' legal authorized representatives took place between the scheduled visits and after study completion. The visits during the trial included physical examination, assessments of developmental functioning (Bayley III scores), kidney ultrasound imaging, blood sampling for safety, PK and biomarkers, assessment of safety, and compliance (see Supplementary Tables S1 and S2 for schedule of trial procedures at screening and follow-up visits).

The study complied with the International Council for Harmonisation of Technical Requirements for Pharmaceuticals for Human Use Guideline for Good Clinical Practice (June 1996), the ethical principles outlined in the Declaration of Helsinki, and all applicable local regulatory and legal standards. Approval for the study protocol was granted by the appropriate Institutional Review Board or Independent Ethics Committee. All participants, or their legal guardians provided written informed consent prior to participation.

### 2.3 Patients

The pediatric substudy was open to male and female patients aged 6 to <24 months, with a diagnosis of NPC1 or NPC2 that was either genetically confirmed (DNA sequence analysis) by pathogenic variants in both alleles of *NPC1* or *NPC2*, or by a pathogenic variant in only one allele of *NPC1* or *NPC2* plus either positive filipin staining, or elevated cholestane triol/oxysterols (>2 × upper limit of normal [ULN]).

Patients receiving prescribed treatment with miglustat needed to be on a stable dose for at least 1 month prior to enrolment. It was recommended that patients did not start miglustat during the first 12 months of arimoclomol exposure in order to minimize potential confounding. Patients with elevated transaminases >3 × ULN, renal insufficiency, with known causes of active liver disease or prolonged icterus or malformation of organs or post liver transplants, or whose clinical condition, in the investigator's opinion, would not allow for the required blood collection as per the protocol-specified procedures were excluded.

### 2.4 Dosing regimen and administration

For patients aged 6 to 23 months, a solution of 3.1 mg/mL arimoclomol concentration was used. This can be prepared by dissolving the content of a 47 mg capsule into 15 mL water. The patient's dose in mL was calculated from the patient's body weight in kg (measured with 1 decimal) by using the following calculation: dose in mL = bodyweight (kg with 1 decimal) * 0.64 mL/kg. The dosing volume was rounded to the nearest 0.2 mL. The solution was administered orally using a 10 mL syringe with 0.2 mL graduations. The measured solution could be added to a small amount (15 mL) of suitable beverage (e.g., water, apple juice) or soft food (e.g., apple sauce, pudding, or yoghurt) and was to be consumed or administered via a feeding tube immediately.

Based on population PK simulations, a starting dose corresponding to 2 mg arimoclomol /kg body weight three times a day (t.i.d.) was selected. This was predicted to result in a mean exposure to arimoclomol citrate over the first 8 h following a dose at steady state (AUC_0–8,SS_) of 2400 h∙μg/L, which is comparable to the mean exposure observed in NPC patients 2–19 years of age (mean AUC_0–8,ss_ of 2600 h∙μg/L; unpublished data). If the initial PK sample demonstrated that a dosing adjustment was needed, the adjusted dose was applied in the above equation going forward. Once patients turned 24 months of age, arimoclomol dosing was changed to the dosing regimen of the main study, based on body weight (Supplementary Table 3) [8].

After the first arimoclomol dose at visit 2, two PK blood samples were drawn 6–8 h after dose administration, with minimum 0.25 h between the two samples (as determined based on population PK simulations) for evaluation of arimoclomol plasma exposure levels. The PK evaluation was preferably performed before visit 3. If the AUC_0–8,SS_ was outside the target exposure, a new dose level was recommended. Any dose adjustment was done at the discretion of the investigator taking into consideration the medical condition of the patient.

During the trial, the principal investigator could consider a (temporary) dose reduction for patients who had serum creatinine above 1.5 × the patient's baseline creatinine value confirmed by a repeated measurement and for patients >2 years showing a decrease in weight meeting the lower weight range in the actual dose group. Dosing could be interrupted if a patient experienced a clinically significant and/or unacceptable toxicity. Administration of arimoclomol had to be stopped and the patient withdrawn from the study if Common Terminology Criteria for Adverse Events (CTCAE) Grade 2 acute kidney injury or any CTCAE Grade 3 adverse events (AEs) considered related to arimoclomol occurred.

During the trial, patients remained on their standard prescribed therapy, symptomatic medication, vitamins, minerals, and food supplements.

### 2.5 Primary outcome

The primary outcome of the study was safety and tolerability of arimoclomol, including AEs, hematology, clinical chemistry and vital signs. Information collected on AEs included duration, severity (according to CTCAE), relationship to arimoclomol, outcome, concomitant therapy given, and action taken with respect to arimoclomol. Serious adverse events (SAEs) were evaluated with respect to seriousness, causality, and expectedness.

### 2.6 Secondary outcomes

Secondary outcomes were clinical status outcomes, including physical examination and change in developmental status, PK, and biomarkers.

Developmental status was assessed by the Bayley Scales of Infant Development Third Edition (BSID-III), a widely used tool for evaluating child development in children aged 1–42 months [15]. The BSID-III was selected for its broad applicability, the availability of growth scale values for assessing change over time independent of chronological age, and the inclusion of nonverbal items on the cognitive scale. The BSID-III assessment was also considered applicable for pediatric substudy patients older than 42 months, as justified by the significant developmental delay in the patient population, including all domains monitored by the scoring tool. The test was administered by trained professionals who had appropriate experience and training. Collected data were evaluated by a Bayley expert for this population (one central reviewer for all sites).

The BSID-III consists of scores in five domains of function in infants: cognitive, communication, physical, social/emotional, and adaptive behavior. Subtest scores for cognitive, receptive communication, expressive communication, fine motor, and gross motor, as well as composite scores for cognitive, language and motor domains were recorded separately. Subtests yield scaled scores with a mean of 10 and a standard deviation of 3. Scaled scores >1 standard deviation below the mean are suggestive of delays compared to age expectations. In addition to scaled scores, person ability scores (BSID-III growth score values, GSVs) were collected to provide a measure of within-person developmental change that is better suited to populations with significant delays [16]. While scaled scores are useful for comparing performance to chronological age expectations, they are limited by floor effects and their inability to reliably detect within-person change over time. A decline in scaled scores does not necessarily represent a decline in ability but may reflect slower developmental progress relative to age-matched peers. GVSs provide an alternative measure that captures individual developmental change over time and have demonstrated statistical superiority to norm-referenced scores for use in clinical trials with patients with significant neurodevelopmental disabilities [16]. Reporting both scaled scores and GSVs allows assessment of patient performance relative to age expectations and evaluation of change in developmental status over time. Change in GSVs over time was calculated at each available time point relative to the individual's baseline GSV score.

For the PK analysis, AUC_0–8,SS_ was determined from samples collected at visit 2 (baseline) to assess if the exposure level was acceptable and to describe PK in patients aged 6 to <24 months at study enrolment. Additional PK data collected at visits 6 (6 months) and 8 (12 months) were incorporated into a population PK model. At visit 2, PK sampling to confirm dose selection was performed at two time-points within the time window of 6–8 h following the first dose of arimoclomol with a minimum 0.25 h between the 2 samples. PK sampling had to be performed prior to the second dose of arimoclomol. At visits 6 and 8, PK samples for population PK were taken 5 min (±5 min) prior to the first daily dose of arimoclomol and 30 min (±5 min) following arimoclomol dosing.

Biomarker assessments, including cholestane-triol (oxysterol) and heat shock protein 70 (HSP70), were explorative. Arimoclomol has been suggested to act via upregulation of CLEAR gene expression which raises CLEAR network protein levels including NPC1 and HSP70 [6]. Elevated plasma cholestane-triol levels serve as a biomarker for NPC, arising from activation of an alternative cholesterol metabolic pathway [17]. Blood samples for analysis of biomarkers were collected according to the schedules in Supplementary Tables S1 and S2.

### 2.7 Statistical analysis

Because of the low number of patients in the study, no formal statistical calculation of sample size was performed. The presentation of data is based on listings of observed data; only AEs were summarized. The safety analysis population was defined as all patients who received any dose of arimoclomol.

## 3 Results

### 3.1 Patient population

The pediatric substudy began on December 19, 2018 (first person, first visit) and was completed on October 31, 2024. Four sites in four countries (Denmark, Germany, United Kingdom, and United States) took part in the study.

Among six patients screened for eligibility, five were enrolled in the study, of which four participated for more than 12 months (Table 1). Two patients completed the study and three were withdrawn prior to the last planned study visit. Two patients were withdrawn after 2.8 years and 1.3 years, respectively, due to withdrawal of consent by the legal authorized representative; the third patient was withdrawn after 4.2 months because criteria for discontinuation of treatment due to AEs were met (treatment interruption exceeding 4 weeks).

**Table 1:** Patient disposition, demographics, and baseline characteristics.

Patient identifier	1	2	3	4	5
Days in study	1029	1116	897	483	128
Duration of arimoclomol exposure (days)	788	1109	886	456	72
Completer/withdrawal at end of study	Withdrawal	Completer	Completer	Withdrawal	Withdrawal
Reason for withdrawal	Informed consent withdrawn	–	–	Informed consent withdrawn	Met stop criteria for arimolclomol administration
*Demographics and baseline characteristics*					
Age (months)	23	14	20	16	19
Time since NPC diagnosis (days)	–a	55	420	461	433
Miglustat use	Yes	Yes	Yes	Yes	Yes
Sex	Female	Female	Female	Male	Male
Race	White	Asian	White	White	Asian
Height (m)	0.87	0.74	0.81	0.80	0.79
Weight (kg)	11.8	7.65	9.70	9.97	10.3
BMI (kg/m^2^)	15.6	14.0	14.8	15.6	16.5
Concomitant disease	–	Decreased blood iron	Splenomegaly, dyslipidemia, gaze palsy, eosinophilia	Splenomegaly, microcytic anemia, gross motor delay, developmental speech disorder	Hepatosplenomegaly
Signs of CNS abnormalities and developmental delay	Physical exam: increased muscle reflexes in both legs, delayed development; Bayley III: cognitive, communication, fine motor and gross motor delay	Physical exam: general hypotonia; Bayley III: Cognitive, communication, and gross motor delay	Physical exam: abnormal supranuclear gaze saccades; Bayley III: no develomental delay^b^	Physical exam: hypotonia; Bayley III: communication and gross motor delay	Physical exam: no CNS abnormalities; Bayley III: mild cognitive delay
*Genotype (allele 1/2)*					
Pathogenic variants	c.3425 T > C/c.3425 T > C	c.2972delA/c.2972delA	c.2932C > T/c.3229C > T	c.1628C > T/c.3107C > T	c.2974G > T/c.2974G > T
Proteins	p.Met1142Thr/p.Met1142Thr	p.Gln991fs/p.Gln991fs	p.Arg978Cys/p.Arg1077Ter	p.Pro543Leu/p.Thr1036Met	p.Gly992Trp/p.Gly992Trp
Variant types	Missense/missense	Frameshift/frameshift	Missense/nonsense	Missense/missense	Missense/missense

NA: not available; NPC: Niemann-Pick disease type C.

a Date of diagnosis of patient 1 was only reported as month and year, thus the exact age at diagnosis and NPC duration could not be calculated. The approximate age at diagnosis was 4–5 months and the approximate NPC duration was 19–20 months.

b Patient 3 showed no developmental delay at baseline but did show a delay before 2 years of age. Therefore this patient can be considered to have early-infantile NPC.

### 3.2 Demographics and baseline characteristics

The five patients (three females, two males) were between 14 and 23 months old at screening. All patients had a confirmed diagnosis of NPC1. Molecular analysis identified previously reported pathogenic variants in both alleles of all five patients. The majority of these pathogenic variants were missense mutations (Table 1). Three patients were homozygous.

Medical history (i.e., conditions that started prior to the start of the substudy) was reported for all five patients. The most frequent medical history findings by Preferred Term (PT) were splenomegaly or hepatosplenomegaly (reported for patients 3, 4, and 5), jaundice (reported for patients 4 and 5), and pyrexia (reported three times for patient 3). Four patients had ongoing concomitant diseases at baseline (Table 1). All patients except patient 5 presented with evident neurological symptoms at baseline. Communication and gross motor delays were reported for three patients (patients 1, 2, and 4). Based on central nervous system evaluations and Bayley-III results, patients 1, 2, 3 and 4 were classified as having the early-infantile form of NPC (onset of neurological symptoms before 2 years of age). Patient 3 was classified as having late-infantile NPC.

All patients received miglustat during the trial; in four miglustat treatment was initiated at least one month prior to enrolment in the study and continued throughout. The remaining patient (patient 4) commenced miglustat treatment 11 months prior to enrolment, continued for 13 months and stopped treatment two months after study enrolment. All patients received other concomitant medications, the most reported being treatment for fever and pain and antibiotics for infectious diseases. Use of anticonvulsants and antispasmodics was reported for patient 1; use of proton pump inhibitors for treatment of reflux was reported for patient 2.

All five patients were exposed to arimoclomol during the substudy. The duration of exposure ranged from 72 to 1109 days (Table 1).

### 3.3 Safety evaluation

AEs relevant for reporting in the substudy were defined as AEs with onset date or date of worsening on or after the date of first intake of arimoclomol. Therefore, all AEs reported can be considered treatment-emergent.

A total of 108 AEs were reported for the five patients. Most AEs were non-serious (86.1%), and mild or moderate in severity (toxicity grade 1 and 2, 90.7%). Most AEs (88%) resolved during the study (Table 2). AEs reported more than once are summarized by system organ class (SOC) and PT in Table 3. Most AEs were in the SOCs Infections and infestations (27.8%), Gastrointestinal disorders (20.4%), General disorders and administration site conditions (15.7%), Respiratory, thoracic and mediastinal disorders (10.2%), and Investigations (including increases in liver enzymes; 8.3%). The most frequently reported AEs were pyrexia (11 AEs in two patients), vomiting (nine AEs in three patients), cough (eight AEs in two patients), nasopharyngitis (7 AEs in two patients), upper respiratory tract infection (five AEs in two patients), and diarrhea (four AEs in three patients).

**Table 2:** Summary of adverse events.

	Arimoclomol (*N* = 5)		
	n	%	E
AEs	5	100	108
SAEs	2	40	15
Non-serious AEs	5	100	93
Fatal AEs	0	0	0
*Toxicity*			
Grade 1	4	80	71
Grade 2	4	80	27
Grade 3	1	20	10
*Relationship*			
Probably related	1	20	2
Unlikely related	1	20	7
Not related	4	80	99
*Action taken with arimoclomol*			
Dose not changed	4	80	106
Drug withdrawn	1	20	2
*Outcome*			
Not recovered/not resolved	4	80	10
Recovered/resolved with sequelae	1	20	1
Recovering/resolving	2	40	2
Recovered/resolved	5	100	95

N: number of patients in the safety analysis set; n: number of patients with event; %: percentage of patients with event; E = number of events; AE: adverse event; SAE: serious adverse event.

**Table 3:** Adverse events by SOC and PT (>1 event reported).

	Arimoclomol (N = 5)		
	n	%	E
*Infections and infestations*	4	80	30
Nasopharyngitis	2	40	7
Upper respiratory tract infection	2	40	5
Pneumonia	1	20	2
*Gastrointestinal disorders*	3	60	22
Vomiting	3	60	9
Diarrhea	3	60	4
Constipation	2	40	3
Tongue ulceration	1	20	3
*General disorders and administration site conditions*	3	60	17
Pyrexia	2	40	11
Disease progression	1	20	2
*Respiratory, thoracic and mediastinal disorders*	3	60	11
Cough	2	40	8
*Investigations*	3	60	9
Alanine aminotransferase increased	3	60	3
Aspartate aminotransferase increased	2	40	3
*Skin and subcutaneous tissue disorders*	2	40	4
*Ear and labyrinth disorders*	2	40	3
*Blood and lymphatic system disorders*	2	40	2
*Metabolism and nutrition disorders*	1	20	2

N: number of patients in the safety analysis set; n: number of patients with event; %: percentage of patients with event; E: number of events; SOC: system organ class; PT: preferred term.

Ten severe AEs (SAEs, toxicity grade 3) were reported for patient 2; three of these were reported as vomiting of 15–22 days duration. All other severe AEs were single occurrences but with temporal overlap between some events (e.g., influenza, vomiting and dehydration occurring within 14 days). All severe AEs required prolonged hospitalization and were therefore also reported as SAEs; all recovered/resolved without changes to arimoclomol. No severe AEs were reported for the other four patients.

A total of 15 SAEs were reported for two patients. These included the three severe AEs of vomiting discussed above and two SAEs of pyrexia, which were each reported for one patient. The remaining SAEs were single occurrences. None of the SAEs were considered related to arimoclomol and all recovered/resolved without dosing changes. No fatal AEs or deaths were reported.

Two AEs, reported for patient 5 on day 36 after first administration of arimoclomol, were considered probably related to arimoclomol. These were moderate AEs of increased alanine aminotransferase and aspartate aminotransferase which resolved after 51 days. As the interruption in arimoclomol treatment exceeded the protocol-allowed 4-week treatment interruption, the patient was withdrawn from the substudy on day 128.

No clinically significant abnormal hematology results were reported. Two AEs related to hematology parameters were reported for patients 2 (moderate anemia) and 3 (moderate eosinophilia). All five patients experienced ≥1 occurrence of elevated, not clinically significant, aspartate aminotransferase levels post screening. Patients 1, 2 and 5 also experienced ≥1 occurrence of elevated, not clinically significant, alanine aminotransferase levels. No clinically significant findings from kidney ultrasound imaging were observed for any of the patients. There were no significant changes in vital signs.

### 3.4 PK analysis

AUC_0–8,SS_ was determined from PK samples at visit 2 (baseline) to assess if the exposure level was acceptable and to describe PK in patients aged 6 to <24 months at study enrolment. Patients 2 and 3 had their arimoclomol dose increased due to the PK assessment at visit 2. Additional PK data collected at 6 and 12 months were incorporated into the population PK model.

Simulations and the population PK model structure were used to calculate AUC_0–8,SS_, which ranged from 1378.3 to 2988 h∙μg/L for the five patients. This range is comparable to the mean exposure observed in NPC patients 2–19 years of age (mean AUC_0–8,ss_ of 2600 h∙μg/L) and demonstrates that the dosing used in the pediatric substudy was appropriate. The population PK model's ability to predict arimoclomol concentrations for each patient was assessed with a range visual predictive check which compared the observed data with simulated concentration-time profiles for 1000 virtual patients of equal age and weight profiles for the study duration. For patients 2, 4 and 5, simulations from the model were consistent with observed data. For patients 1 and 3 the observations were below the lower limit of the 90% prediction interval at visit 2; improved consistency between predictions and observations were seen at later visits.

### 3.5 Bayley III scores

**Fig. 2:**
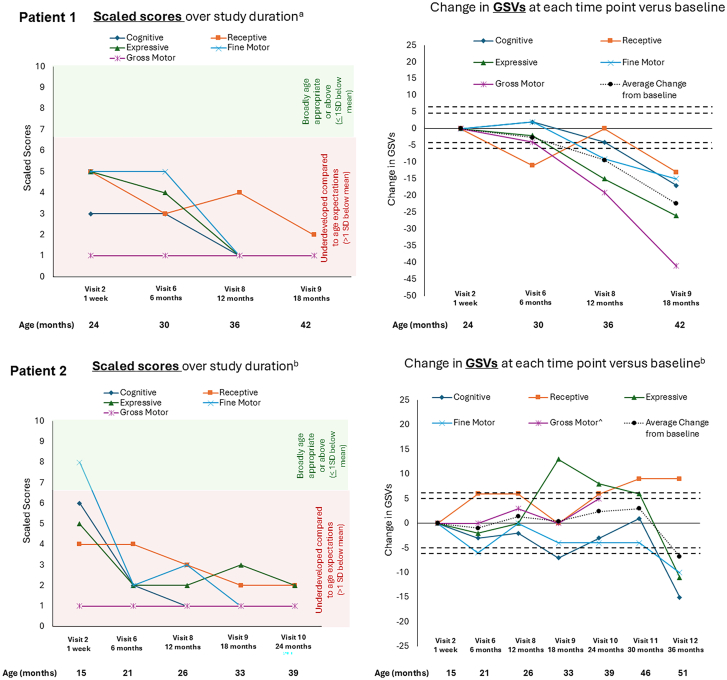

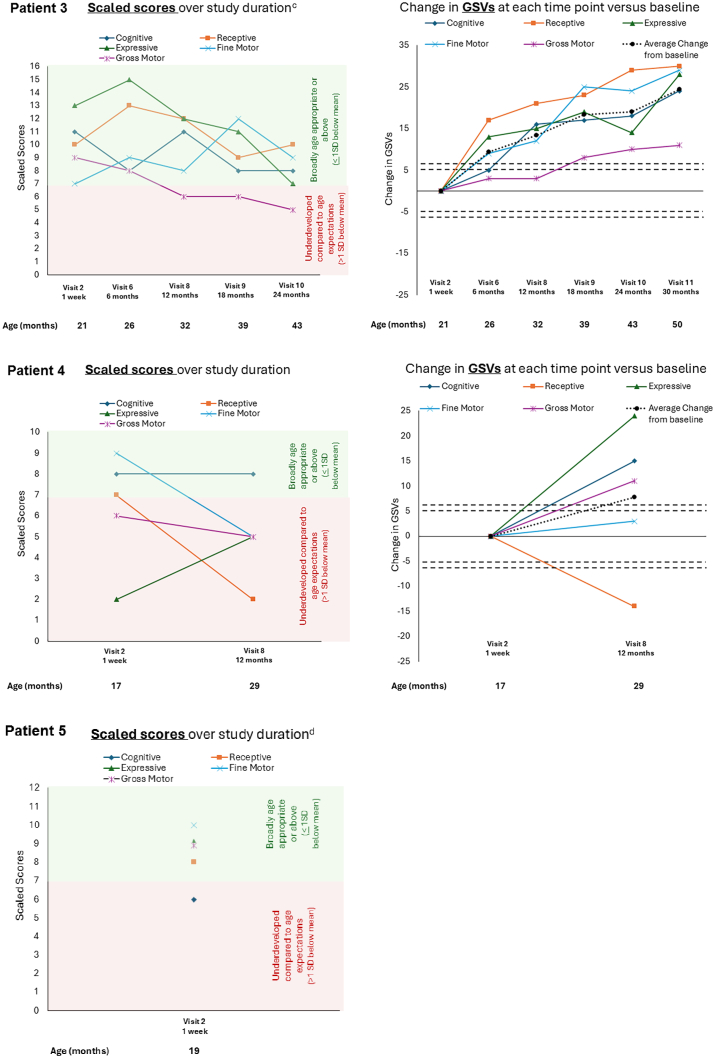
Bayley III results over the course of the study. ^a^ Patient 1: lines for cognitive, expressive, fine motor, and gross motor overlap for visit 8 to 9 with scaled scores of 1 across these domains at both of visits. ^b^ Patient 2: no scaled score data available for visits 11 or 12 because patient was out of age range and thus standard scores not available. Gross motor not administered for this participant at visits 11 or 12. Patient was not able to fully participate at visit 12 due to tiredness. ^c^ Patient 3: no data available for visit 11 because participant was out of age range and thus standard scores not available. ^d^ Patient 5: change in GSVs not calculated as only one data point available.

Individual Bayley III subtest scores are presented in Fig. 2. Both norm-reference scores (BSID-III scaled scores) and person ability scores (BSDI-III GSVs) are reported to allow assessment of patient performance relative to age expectations and evaluation of change in developmental status over time.

Scaled Bayley III subtest scores (indicating how the individual performed at each time point compared to age expectations) of patient 1 showed development below average for the cognitive, expressive communication, receptive communication, fine motor and gross motor domains at baseline. Scaled scores declined for all domains, except gross motor due to floor effects, during the study (Fig. 2). In contrast, changes in GSVs, indicating how the individual performed compared to her own baseline performance, showed largely stable cognitive, expressive communication, fine motor and gross motor domain scores from baseline to 6 months though subsequent declines in all domains through 18 months of treatment. Receptive communication scores showed a more variable pattern.

Scaled scores of patient 2 were below average for the cognitive, expressive communication, receptive communication, and gross motor domains at baseline. Fine motor abilities were within age expectations at baseline. Scores declined for all domains, except gross motor due to floor effects, during the study (Fig. 2). However, changes in GSVs showed stability across all domains from baseline to 30 months, and slight improvements in expressive and receptive communication from 18 to 24 months of treatment, respectively. The recording of substantial declines seen between 30 and 36 months across all domains, except receptive communication, were considered an artifact (due to tiredness of the patient underperforming on testing) rather than a meaningful regression. Overall, analysis of GSVs suggested stable developmental function during the study.

Scaled scores of patient 3 indicated broadly age-appropriate developmental function for all domains assessed. The gross motor domain showed a mild and gradual decrease in scaled scores, falling below age expectations at 12 through 24 months of treatment (Fig. 2). However, changes in GSVs indicated consistent improvements across all domains during the study compared to baseline levels, indicating improvements in developmental skills.

Baseline scaled scores of patient 4 indicated age-appropriate abilities for the cognitive, receptive communication and fine motor domains, and underdeveloped abilities in the expressive communication and gross motor domains. Follow-up data were only available for 12 months (Fig. 2). Changes in GSVs between these visits indicated modest improvements in the cognitive domain, marked improvements in expressive communication and gross motor domains, stability in the fine motor domain and a decline in the receptive communication domain, suggesting largely stable and potentially improved development during this period across most domains.

Scaled scores of patient 5 suggested average abilities in the expressive communication, receptive communication, fine motor and gross motor domains though underdeveloped cognitive functioning compared to age expectations at baseline (Fig. 2). Since only baseline Bayley III scores were collected, change in GSVs over time could not be calculated.

### 3.6 Biomarkers

Cholestane-triol increased from baseline to last assessment in patients 1, 2 and 3 (range: 10.7–44.23 μg/L for observations spanning 555–1116 days) and decreased from baseline to last assessment in patients 4 and 5 (−18.77 and − 41 μg/L after 482 and 128 days, respectively) (Supplementary Table S4).

HSP70 increased from baseline to last assessment in patients 1, 2 and 4 (range: 354.91–3032.16 ng/L for observations spanning 482–1116 days). Patient 3 did not have a baseline assessment, however an increase in HSP70 (395.48 ng/L) was seen from an assessment at a later visit (6 months) to the last assessment (30 months), 716 days apart. For patient 5, HSP70 decreased from baseline to last assessment (−215.42 ng/L), just 5 days apart.

## 4 Discussion

The pivotal NPC-002 study previously demonstrated a statistically significant and clinically meaningful reduction in disease progression in a heterogeneous population of NPC patients receiving arimoclomol in addition to miglustat [8]. This reduction was sustained for at least 5 years during the open-label extension of the trial [9]. Arimoclomol was well tolerated. However, the primary NPC-002 study did not include patients below 2 years of age. The present prospective, open-label, multicenter, pediatric substudy provides insight into the safety, tolerability, and PK of arimoclomol in infants with NPC aged 6 to <24 months at study enrolment for up to 36 months of treatment.

Overall, the five study participants' medical histories, concomitant medications, and clinical examination findings were consistent with their underlying NPC disease and young age, while also highlighting notable clinical heterogeneity. Some participants presented with multiple comorbidities and/or developmental delays at baseline, whereas others appeared to be less severely affected. Based on central nervous system evaluations and Bayley-III results, four patients (patients 1, 2, 3 and 4) can be classified as having the early-infantile form of NPC, characterized by neurological symptom onset before 2 years of age. The observed phenotypic heterogeneity among participants is in line with their genotypic variability. All identified variants have previously been classified as pathogenic or likely pathogenic (https://www.ncbi.nlm.nih.gov/clinvar/).

During the substudy, all five patients were exposed to arimoclomol for 72 days to 1109 days (∼3 years) alongside standard clinical care, including miglustat. Four patients received arimoclomol for more than a year. Overall, arimoclomol appeared to be well tolerated by the study population, with no new safety signals identified. The AEs and safety parameters observed were consistent with previous findings [8]. The most frequently reported AEs were pyrexia, vomiting, cough, nasopharyngitis, upper respiratory tract infection, and diarrhea, with most AEs being mild to moderate in severity and resolving without requiring interruption of treatment. No hematological parameters were deemed abnormal or clinically significant, and kidney ultrasound imaging revealed no clinically relevant findings. All patients experienced fluctuations in transaminase levels exceeding the reference range at some point during the trial, though none were considered clinically significant. It is worth noting that elevations in transaminase levels are not uncommon in patients with NPC, given the frequent involvement of the liver [5], [12], [18]. The two treatment-related AEs of increased alanine aminotransferase and aspartate aminotransferase that led to treatment discontinuation of patient 5 resolved after cessation of arimoclomol treatment; however, these adverse events may rather reflect underlying disease pathology, as elevated hepatic enzymes are commonly observed in NPC.

The PK analysis showed a AUC_0–8,SS_ for the five patients in the substudy ranging from 1378.3 to 2988 h∙μg/L. This range is comparable to the mean exposure observed in NPC patients 2–19 years of age (mean AU_C0–8,SS_ of 2600 h∙μg/L; unpublished data), indicating that the dosing used in the substudy was appropriate.

Bayley III test data collected at baseline and follow-up provided insight into developmental delays among the study population and helped characterize each patient's phenotypic severity. All patients showed delays compared with age expectations in at least one domain, with gross motor function most frequently affected. Two patients exhibited poor performance compared with age expectations across nearly all domains. Changes in growth scale values indicated that one patient showed developmental gains, two remained largely stable, and one experienced a general decline. For the fifth patient, only baseline data were available, precluding evaluation of change over time. It should be noted that, given the small sample size and the uncontrolled design of the study, these findings cannot be used to draw conclusions regarding the therapeutic efficacy of arimoclomol in this population in terms of developmental status.

Biomarker analyses yielded variable results, with cholestane-triol and HSP70 levels decreasing in some patients and increasing in others. This is not unexpected given the exploratory nature of these assessments. The reason for the variability in biomarker responses between the participants of the study remains unclear and requires further investigation. To date, no biomarker has demonstrated a consistent correlation with NPC severity, highlighting the need for validated, sensitive biomarkers for NPC disease to evaluate progression and therapeutic response in clinical trials [19]. Due to the limited amount of blood that can be safely collected from infants, it was not possible to explore additional biomarkers in the study.

A key limitation of this substudy is its very small sample size, which precludes drawing firm conclusions regarding the safety, tolerability, or efficacy of arimoclomol in infants with NPC. Nevertheless, the data provide valuable first insights and may serve as a preliminary basis for future investigations in larger patient cohorts. An additional potential limitation is that four patients initiated miglustat less than 6 months before starting arimoclomol, which could be attributed to their young age and short time since diagnosis. Based on data from the pivotal study demonstrating that miglustat and arimoclomol can be safely co administered, the protocol allowed miglustat to be introduced approximately 1 month before arimoclomol. The design of the substudy in infants reflected a deliberate balance: ensuring that these vulnerable children could receive both treatments as early as possible, while acknowledging that introducing miglustat shortly before baseline might influence safety and efficacy assessments. Notably, previous studies have shown only limited clinical benefit of miglustat in NPC patients with a similar clinical profile, further supporting that the observed outcomes are unlikely to be driven by miglustat only [13], [14].

## 5 Conclusions

The results of the pediatric substudy provide evidence that arimoclomol may be well tolerated in infants when treatment was initiated before 2 years of age, with no new safety signals observed. PK findings confirmed that the dosing regimen used in the pediatric substudy was appropriate. These findings suggest that early initiation of arimoclomol could be considered for the 6–24-month population. However, additional exposure date is needed to accurately characterize the safety and tolerability profile for this subset of patients.

A plain-language summary of the study is included in the supplementary materials.

## CRediT authorship contribution statement

**Eugen Mengel:** Writing – review & editing, Investigation, Formal analysis, Data curation, Conceptualization. **Laila Arash-Kaps:** Writing – review & editing, Investigation, Data curation. **Stephanie Grunewald:** Writing – review & editing, Investigation, Data curation. **Sabine Weller Grønborg:** Writing – review & editing, Investigation, Data curation. **Natalie Berger:** Writing – review & editing, Formal analysis. **Hadeel Shammas:** Writing – review & editing, Formal analysis. **Christine í Dali:** Writing – review & editing, Methodology, Formal analysis, Conceptualization.

## Ethics approval and consent to participate

The trial (ClinicalTrials.gov identifier: NCT02612129) protocol and associated documentation were approved by the relevant independent ethics committees and/or institutional review boards, and written informed consent was obtained at enrollment from either the patient or their legal guardian.

## Funding

The research was funded by Zevra Therapeutics Inc., Copenhagen, Denmark, who were involved in all stages of the trial design, data collection, analysis, and interpretation of the results.

## Declaration of competing interest

Eugen Mengel has received investigator fees and/or consultant honoraria from Cyclo Therapeutics, Amicus, Idorsia, Intrabio, Denali, JCR, Prevail, Freeline Therapeutics, Alexion, Zevra, Sanofi Genzyme, and Takeda.

Laila Arash-Kaps received honoraria/travel grants from Takeda, Zevra, Amicus, and Genorph.

Stephanie Grunewald has received consultancy funding from Hyperion, Origin, Moderna, Nutricia, Sobi, Glycomine, and Ultragenyx, and has participated in commercially funded research and received travel grants from Zevra.

Sabine Weller Grønborg has received travel expenses and congress fee reimbursements from Sanofi Genzyme, participated in Orchard Therapeutics advisory board and sponsored meetings, received speaker honoraria from Actelion and Novo Nordisk, and consultancy honoraria from Immedica Pharma.

Natalie Berger received consulting fees from Praxis Precision Medicine and Zevra Therapeutics Inc.

Hadeel Shammas is an employee of Zevra Therapeutics Inc.

Christine í Dali is an employee and shareholder of Zevra Therapeutics Inc.

## Data Availability

The trial protocol and Statistical Analysis Plans will become publicly available. Study information will be posted on https://clinicaltrials.gov/ct2/show/NCT02612129. The data that support the findings of this trial are available from Zevra Therapeutics Inc. but restrictions apply to the availability of these data, which were used under license for the current trial, and so are not publicly available. Data are however available from the authors upon reasonable request and with permission of Zevra Therapeutics Inc.

## Appendix A Supplementary data

Supplementary materials: Table S1. Schedule of trial procedures visits 1-6; Table S2. Schedule of trial procedures visits 7-12; Table S3. Arimoclomol dosing based on body weight in patients aged 24 months or older; Table S4. Cholestane-triol and HSP70 at baseline and last assessment

Plain laguage summary
