# Supplementary material for: Arimoclomol in infants with Niemann-Pick disease type C: Results from the phase 2/3 open-label pediatric substudy
Source: Mol Genet Metab Rep. 2026 Jun 19;48:101332. doi: 10.1016/j.ymgmr.2026.101332 (PMC13310637; doi:10.1016/j.ymgmr.2026.101332)
Supplement: Supplementary materials: Table S1. Schedule of trial procedures visits 1-6; Table S2. Schedule of trial procedures visits 7-12; Table S3. Arimoclomol dosing based on body weight in patients aged 24 months or older; Table S4. Cholestane-triol and HSP70 at baseline and last assessment [file mmc1.pdf]

Supplementary materials

Table S1. Schedule of trial procedures visits 1-6

	Screen	Treatment					Unsch. visit[a]
**Visit**	**1**	**2**	**3**	**4**	**5**	**6**	**NA**
Prodecures	NA	Within 7 days of Visit 1	7-14 days after Visit 2	1 month (±1 week) after Visit 2	3 months (±1 week) after Visit 2	6 Months (±2 weeks) after Visit 2	NA
Informed consent	X						
Inclusion/exclusion criteria	X	X[b]					
Demographics	X						
NPC diagnosis	X						
NPC disease history	X						
Medical history	X						
Physical examination	X	X		X	X	X	(X)
Weight	X	X		X	X	X	(X)
Height	X	X			X	X	(X)
Vital signs	X	X	X	X	X	X	(X)
Hematology & clinical chemistry[c]	X		X	X	X	X	(X)
Blood sample for biomarker analysis[d]	X	(X)[d]				X	
Ultrasound (liver, spleen kidneys)		X				X	
Bayley III score		X				X	
Concomitant therapy[e]	X	X	X	X	X	X	X
Adverse events	X	X	X	X	X	X	X
Arimoclomol dispensing & admnistration		X	X	X	X	X	X
Arimoclomol dose adjustment[f]			(X)	(X)	(X)	(X)	(X)
Arimoclomol: Return[g]			X	X	X	X	(X)
PK sampling[h]		X[h]				X[h]	(X)
Telephone follow-up[i]					X (monthly)		

[a] Unscheduled visit: The patient could attend the site for unscheduled visits if there were any safety concerns, if the patient's weight required reassessment in the clinic (in order to confirm if a dose adjustment is required) or as per investigator discretion. Arimoclomol return and dispensing could take place in the event that a dose adjustment had occurred. Visit assessments (physical examination, weight, height, vital signs, hematology & clinical chemistry) and PK sampling were performed at the discretion of the investigator
[b] Exclusion criteria on central laboratory assessments had to be evaluated prior to dosing the patient
[c] Hematology & clinical chemistry samples were sent to a central laboratory for analysis
[d] Biomarker samples were sent to a central laboratory for analysis. For Visit 2, sample were taken only for patients who weigh ≥8 kg
[e] Concomitant therapy includes all medication and medical procedures (including any unplanned diagnostic, therapeutic or surgical procedures) ongoing at or starting after the time of written consent
[f] Arimoclomol dose was adjusted based on the results of the PK evaluation performed by a PK dosing committee and/or based on serum creatinine values and/or based on a substantial change in the patient's weight (as confirmed during a site visit)
[g] Patients were required to return all unused arimoclomol (and relevant packaging) to the site staff. The site staff had to follow up on the reasons for any missing arimoclomol or other discrepancies noted after performing the relevant arimoclomol accountability
[h] Visit 2: PK sampling was performed at 2 timepoints within the time window of 6 to 8 hours following the first dose of arimoclomol with a minimum 0.25 hours between the 2 samples; Visit 6: 5 min (±5 min) prior to the first daily dose of arimoclomol and 30 min (±5 min) following arimoclomol dosing. The last dose of arimoclomol had to be taken more than 8 hours prior to the site visit. In the event that an unexpected PK evaluation was obtained which is clinically significant and could potentially impact the safety of the patient, the PK dosing committee could recommend additional PK sampling
[i] Telephone follow-up included follow up on the status of the patient, whether the patient has experienced any new adverse events (AEs) or the worsening of any existing AEs, whether the patient has had a change in any prescribed medication, to confirm the weight of the patient and whether the patient has experienced any difficulties in taking arimoclomol.

Table S2. Schedule of trial procedures visits 7-12

								**Follow-up**	**Unsch. visit**[a]
**Visit**	**7**	**8**	**(8a)**[b]	**9**	**10**	**11**	**12/ end of study**	**Telephone call**	
Procedures	9 months (±2 weeks) after Visit 2	12 months (±2 weeks) after Visit 2	15 months (±2 weeks) after Visit 2	18 months (±2 weeks) after Visit 2	24 months (±2 weeks) after Visit 2	30 months (±2 weeks) after Visit 2	36 months (±2 weeks) after Visit 2 or within 4 weeks of withdrawal from study	2 weeks (+2 weeks) following End of Study visit	NA
Physcial examination	X	X	X	X	X	X	X		(X)
Weight	X	X	X	X	X	X	X		(X)
Height		X	X	X	X	X	X		(X)
Vital signs	X	X	X	X	X	X	X		(X)
Hematology & clinical chemistry[c]	X	X	X	X	X	X	X		(X)
Blood sample for biomarker analysis		X		X	X	X	X		
Ultrasound (liver, spleen, kidneys)		X		X					
Bayley III score		X		X	X	X	X		
Concomitant therapy	X	X	X	X	X	X	X		X
Adverse events	X	X	X	X	X	X	X	X[i]	X
Arimoclomol: dispensing & administration	X	X	X	X	X	X			X
Arimoclomol dose adjustment[f]	(X)	(X)	(X)	(X)	(X)	(X)			(X)
Arimoclomol: return[g]	X	X	X	X	X	X	X		(X)
PK sampling[h]		X							(X)
Telephone follow-up[i]	X (monthly)			X (every 3-4 months)				X	

[a] Unscheduled visit: The patient could attend the site for unscheduled visits if there were any safety concerns, if the patient's weight required reassessment in the clinic (in order to confirm if a dose adjustment is required) or as per investigator discretion. Arimoclomol return and dispensing could take place in the event that a dose adjustment had occurred. Visit assessments (physical examination, weight, height, vital signs, hematology & clinical chemistry) and PK sampling were performed at the discretion of the investigator

[b] Visit 8a for pediatric substudy patients aged <12 months at study enrolment

[c] Hematology & clinical chemistry samples were sent to a central laboratory for analysis

[d] Biomarker samples were sent to a central laboratory for analysis

[e] Concomitant therapy includes all medication and medical procedures (including any unplanned diagnostic, therapeutic or surgical procedures) ongoing at or starting after the time of written consent

[f] Arimoclomol dose was adjusted based on the results of the PK evaluation performed by a PK dosing committee and/or based on serum creatinine values and/or based on a substantial change in the patient's weight (as confirmed during a site visit)

[g] Patients were required to return all unused arimoclomol (and relevant packaging) to the site staff. The site staff had to follow up on the reasons for any missing arimoclomol or other discrepancies noted after performing the relevant arimoclomol accountability

[h] Visit 8: 5 min (±5 min) prior to the first daily dose of arimoclomol and 30 min (±5 min) following arimoclomol dosing. The last dose of arimoclomol had to be taken more than 8 hours prior to the site visit

Telephone follow-up included follow up on the status of the patient, whether the patient has experienced any new AEs or the worsening of any existing adverse events (AEs), whether the patient has had a change in any prescribed medication, to confirm the weight of the patient and whether the patient has experienced any difficulties in taking arimoclomol. Additionally, the investigator contacted the legal authorized representative by telephone after the end of study to follow up on the status of the patient and to confirm whether any new AEs, which the investigator assesses as related to a study procedure and/or arimoclomol, have occurred

Table S3. Arimoclomol dosing based on body weight in patients aged 24 months or older

Patient body weight	Arimoclomol citrate dose	Corresponding dose of arimoclomol base[a]	Solution of 100 mg arimoclomol citrate capsule in 20 mL liquid (5 mg/mL)
8-15 kg	50 mg t.i.d. (150 mg/day)	31 mg t.i.d.	10 mL t.i.d.
>15-22 kg	75 mg t.i.d. (225 mg/day)	47 mg t.i.d.	15 mL t.i.d.
>22-38 kg	100 mg t.i.d. (300 mg/day)	62 mg t.i.d.	20 mL t.i.d.
>38-55 kg	150 mg t.i.d. (450 mg/day)	93 mg t.i.d.	30 mL[b] t.i.d.
>55 kg	200 mg t.i.d. (600 mg/day)	124 mg t.i.d.	40 mL[b] t.i.d.

t.i.d.: 3 times per day.
[a] Conversion factor from arimoclomol citrate to arimoclomol free base: [citrate dose] * 0.620241
[b] Two 100 mg arimoclomol citrate capsules are dissolved in 40mL liquid (5 mg/mL)

Table S4. Cholestane-triol and HSP70 at baseline and last assessment

Patient identifier	Cholestane-triol (µg/L)			HSP70 (ng/L)			
	Baseline[a]	Last assessment	Change from baseline to last assessment	Baseline[a]	Last assessment	Change from baseline to last assessment	Time from Baseline to last assessment (days)
1	107.09	145.25	38.16	1970.17	2325.08	354.91	555
2	92.03	136.26	44.23	950.43	3982.59	3032.16	1116
3	65.11	75.81	10.70	ND	3735.86	N/A	897
4	94.64	75.87	-18.77	2211.86	2604.21	392.35	482
5	115.46	74.46	-41.00	3184.57	2969.15	-215.42	128 / 5

HSP70: Heat shock protein 70; N/A = not applicable; ND = not done

[a]Screening (visit 1) was baseline
