# Supplementary material for: Arimoclomol in infants with Niemann-Pick disease type C: Results from the phase 2/3 open-label pediatric substudy
Source: Mol Genet Metab Rep. 2026 Jun 19;48:101332. doi: 10.1016/j.ymgmr.2026.101332 (PMC13310637; doi:10.1016/j.ymgmr.2026.101332)
Supplement: Plain laguage summary [file mmc2.pdf]

Arimoclomol in infants with Niemann-Pick disease type C: Results from the phase 2/3 open-label pediatric substudy

Eugen Mengel; Laila Arash-Kaps; Stephanie Grunewald; Sabine Weller Grønborg; Natalie Berger; Hadeel Shammas; Christine í Dali

Key learnings

1. A pedicatric substudy evaluated the safety of arimoclomol, how it distributes in the body (pharmacokinetics), and its effects in infants with Niemann Pick disease type C (NPC) aged less than 2 years
2. When given along with standard treatment (including miglustat) for up to 3 years, arimoclomol was generally well tolerated, with no new safety concerns found
3. The study supported the dose that was used, indicating that starting arimoclomol in infants could be considered

Background

- **NPC:** A rare genetic condition that leads to progressive damage to the brain and nervous system and often shortening life expectancy
- **Arimoclomol:** An oral medication approved in the US for people with NPC who are 2 years or older, to be used in combination with miglustat

Phase 2/3 open-label pediatric substudy

- 5 infants with NPC (3 boys, 2 girls)
- Aged 14–23 months at screening

- Arimoclomol was given for up to 3 years (72 to 1109 days)
- Arimoclomol was given in addition to standard treatment, including miglustat, in all patients

Primary endpoint

Safety and tolerability of arimoclomolpediatric substudy

Side effects (adverse events): 108 were reported, but most were mild or moderate in severity and not serious

Side effects probably linked to arimoclomol: One patient had temporary increases in liver enzymes (2 events), which returned to normal within 51 days

Blood tests, kidney scans, and vital signs: No important changes were seen

Secondary endpoints

Distribution of arimoclomol in the body: Average levels in the blood during the first 8 hours after dosing were similar to those seen in older children and teenagers with NPC

Development and biomarkers: Measures of development skills showed both improvement, stabilization and decline over time; biological markers showed mixed changes
